# Undiagnosed uterus didelphys with unicavitary twin gestation in a district hospital in Ghana: a case report

**DOI:** 10.11604/pamj.2023.44.205.39546

**Published:** 2023-04-28

**Authors:** Kofi Effah, Lawrencia Serwaa Manu, Emilia Asuquo Udofia, Nana Owusu Essel

**Affiliations:** 1 Cervical Cancer Prevention and Training Centre, Catholic Hospital, Battor, Ghana,; 2 Department of Obstetrics and Gynecology, Catholic Hospital, Battor, Ghana,; 3 Department of Obstetrics and Gynecology, Korle Bu Teaching Hospital, Accra, Ghana,; 4 Department of Community Health, University of Ghana Medical School, College of Health Sciences, University of Ghana, Legon, Accra, Ghana,; 5 Department of Emergency Medicine, College of Health Sciences, Faculty of Medicine and Dentistry, University of Alberta, Edmonton, Canada

**Keywords:** Uterus didelphys, twin gestation, pre-eclampsia, Mullerian duct abnormality, case report

## Abstract

Uterus didelphys is a congenital anomaly of the female reproductive tract which arises from the abnormal fusion of the Mullerian ducts. We present, the first case to the best of our knowledge, of uterus didelphys with a unicavitary twin gestation to be documented in Ghana, a low-middle income country. A 24-year-old woman, gravida 3, para 0+2 miscarriages, was seen and admitted to our maternity ward due to elevated blood pressure with ++ proteinuria at 36 weeks of gestation. She attended an antenatal clinic regularly during the pregnancy but was mainly seen by midwives. Apart from multiple pregnancy, two 2D ultrasound examinations (one at 25 weeks gestation and another during admission) did not reveal any uterine malformations. At 37 weeks+2 days, she underwent emergency cesarean section on account of pre-eclampsia and a twin pregnancy with the leading twin in breech presentation. After delivering both babies and the placenta, the uterus was exteriorized and inspected, during which a non-gravid bulky left uterus was first found. Each uterus had a normal ovary and fallopian tube on its lateral end. Further postoperative examination revealed a normal-looking vulva, two vaginas, and two cervices. Both babies weighed 1.9 kg, each below the fifth percentile of weight for age. The elevated blood settled postoperatively and the postoperative period was uneventful. The patient and twins were found in a stable condition on review two weeks after delivery and the twins were healthy at 5 years. Despite being a rare presentation, we wish to create awareness among health workers in rural and low-resource settings of such cases and highlight the need to improve prenatal diagnostic capabilities, as this is key to determining the mode of delivery and achieving favorable maternal and fetal outcomes.

## Introduction

Twin gestation in uterus didelphys is rare, with an incidence of one in 3000 [1]. It presents a challenge to obstetricians, particularly when not diagnosed prior to labor onset. Although typically asymptomatic, uterus didelphys has been associated with dysmenorrhea, dyspareunia, recurrent miscarriage, fetal malpresentation, prematurity, placenta previa, and abruptio placentae [2]. Where available, 3D transvaginal ultrasound offers an excellent non-invasive approach to its diagnosis [1]. Additional investigative modalities include sonohysterography, hysterosalpingography, laparoscopy, and pelvic magnetic resonance imaging, which may not be readily available in low-resource settings. Due in part to failure of diagnosis before labor, the incidence of cesarean birth in uterus didelphys has been reported to be as high as 82% [3]. Reported cases have mostly shown favorable outcomes, including spontaneous vaginal deliveries, and twin and triplet cesarean deliveries [4]. In this paper, we report a rare case of unicavitary twin gestation in a young Ghanaian woman with uterus didelphys which was incidentally found during a cesarean section performed on account of severe preeclampsia with the leading twin in breech presentation. Here, we emphasize the importance of having a high index of suspicion of uterine anomalies among women presenting with recurrent miscarriages, subfertility, and fetal malpresentation. We further highlight the challenges faced in the diagnosis and management of such cases in resource-limited settings.

## Patient and observation

**Patient information:** a 24-year-old woman, gravida 3, para 0 + 2 miscarriages, was seen and admitted to the maternity ward of Catholic Hospital, Battor, Ghana on account of elevated blood pressure (BP) with proteinuria of (++) at 36 weeks of gestation (calculated from a late ultrasound performed at 25 weeks 3 days of gestation). She attended an antenatal clinic regularly during the pregnancy but was mainly seen by midwives. The pregnancy till then had been uneventful with normal BP measurements and unremarkable laboratory test results. She had no family history of congenital anomalies. She had experienced two uncomplicated miscarriages which were not managed in the hospital; thus, she had not been thoroughly examined.

**Diagnostic assessments:** although the patient presented for her antenatal booking visit at 7 weeks of pregnancy, she had only undergone two obstetric ultrasound scans, both of which were 2D and abdominal. Her first obstetric scan was performed at her seventh antenatal visit at an estimated gestational age (EGA) of 25 weeks + 3 days and her second ultrasound scan was performed during the current admission. Apart from a diagnosis of multiple pregnancy, none revealed any uterine anomaly. Transvaginal and 3D abdominal scans were unavailable.

**Timeline of current episode:** on admission, the patient was pale. A complete blood count revealed moderate anemia, with a hemoglobin level of 8 g/dL, which was managed via whole blood transfusion. Bedside ultrasonography showed twin gestation with the leading twin in breech presentation. On day 2 of admission, her BP increased to 170/120 mmHg; thus, treatment with antihypertensives was initiated. She was diagnosed with preeclampsia and managed according to our institutional protocol. At an EGA of 37 weeks + 2 days, she was prepared for an emergency cesarean section on account of pre-eclampsia and a twin pregnancy with the leading twin in breech presentation.

**Figure 1:**
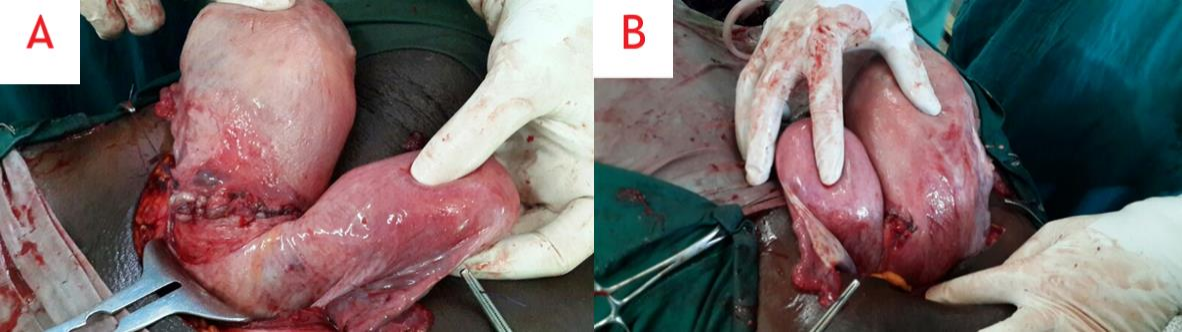
intraoperative images of A) anterior and B) posterior aspects of the exteriorized uterus didelphys with each hemi-uterus having its own fallopian tube and ovary; the smaller, bulky, non-gravid left uterus was located adjacent to the lower uterine segment incision made on the right uterus

**Figure 2:**
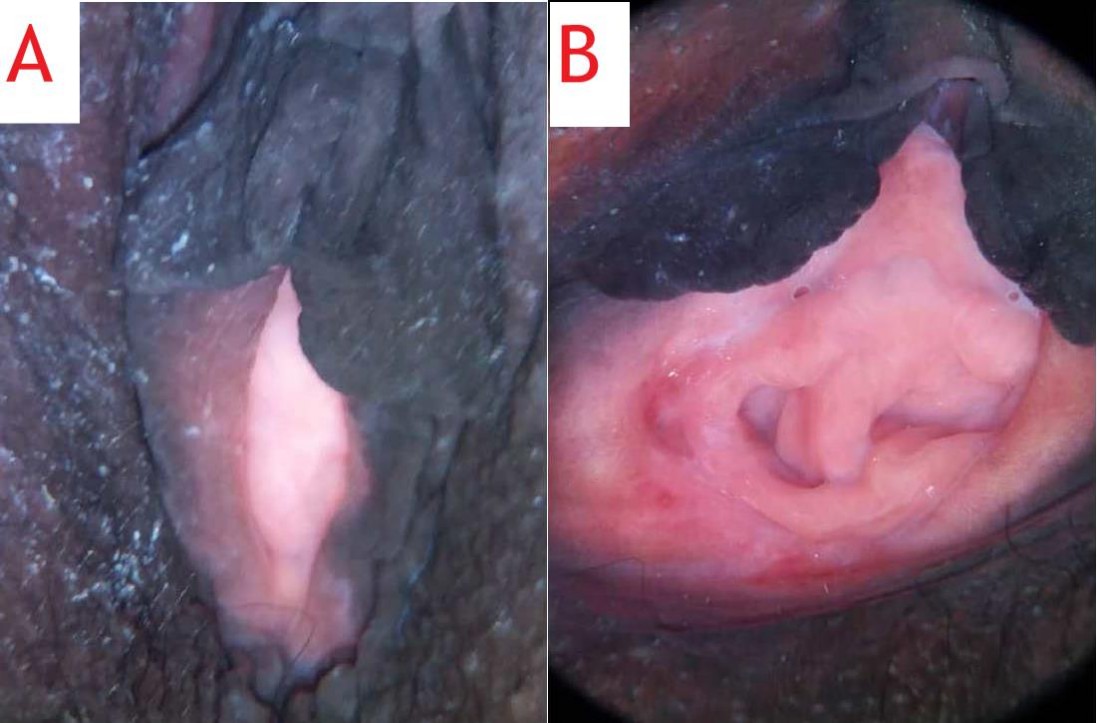
postoperative pelvic images of the patient showing A) a normal vulva; B) labia parted revealing two vaginas with a non-communicative longitudinal vaginal septum

**Figure 3:**
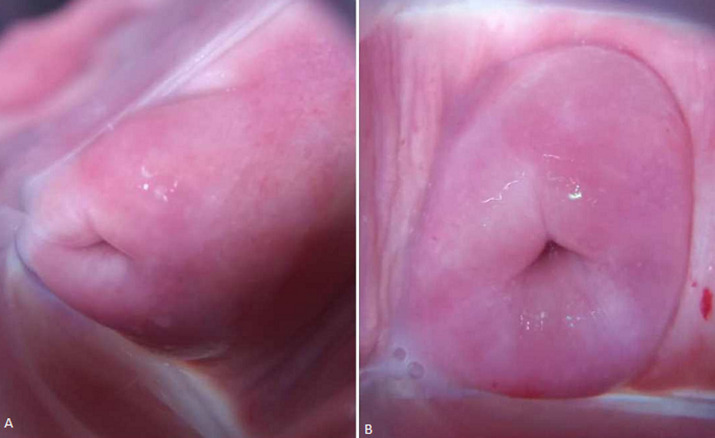
Postoperative pelvic images of the patient showing the A) left and B) right cervices of the uterus didelphys

**Therapeutic interventions:** under spinal anesthesia, the abdomen was entered via a Pfannenstiel incision and the right uterus containing both babies was entered through a lower segment transverse incision. A set of dichorionic, diamniotic, live male twins was delivered. The appearance, pulse, grimace, activity, and respiration (APGAR) scores were 9 and 9 at 1 and 5 minutes, respectively, for the leading twin and 7 and 9 at 1 and 5 minutes, respectively, for the second twin. The first twin was in breech presentation while the second twin had a cephalic presentation. The placentas were located in the anterofundal aspect of the right uterus. After delivering both babies and the placenta, the uterus was exteriorized and inspected. It was then that a non-gravid left uterus was first found and observed to be slightly bulky (Figure 1). Each uterus had a normal ovary and fallopian tube on its lateral end. Further examination revealed a normal-looking vulva, two vaginas (Figure 2), and two cervices (Figure 3).

**Diagnosis:** uterus didelphys with unicavitary twin gestation

**Follow-up and outcomes of interventions:** both babies weighed 1.9 kg, each below the fifth percentile of weight for age. Maternal renal function tests showed normal findings and abdominal ultrasound did not reveal any renal anomalies. The elevated BP had settled postoperatively, and the postoperative period was uneventful. The patient and her babies were discharged on postoperative day 8 after counseling her on family planning and the impact of the findings on her future pregnancies. The patient and twins were found in a stable condition on review two weeks after delivery and the twins were healthy at 5 years. On cervical screening with GeneXpert HPV DNA testing five years after delivery, both cervices tested negative for high-risk human papillomavirus (HPV).

**Patient perspective:** the patient was satisfied with the care she received during her antenatal visits and during and after delivery.

**Informed consent:** written informed consent was obtained from the patient for the publication of this case report and its accompanying images.

## Discussion

Uterus didelphys with twin pregnancy is a very rare presentation. It occurs following abnormal fusion or canalization of the Mullerian ducts during the fetal period. The prevalence of uterus didelphys in Ghana is currently not documented; however, it has a global prevalence of 5.5-6.6% in the general population [5]. The highest prevalence has been reported in women with subfertility (7.3%) and recurrent spontaneous abortions (16.7%) [6]. Multiple gestation in women with uterine malformations leads to a significantly increased risk of maternal and fetal complications in excess of those seen among women without malformations [7]. These include malpresentation, uterine rupture, low birth weight, and dynamic dystocia. Despite these complications, many such patients have experienced uneventful pregnancies and uncomplicated deliveries [8]. Our patient had two previous miscarriages prior to the current pregnancy. She was able to carry the pregnancy to term and had two live births, although both babies were small for gestational age. Both babies had weights below the 5^th^ percentile for their EGA of 37 weeks, indicative of growth restriction. If the leading twin had a cephalic presentation, there would have been the option of induction of labor. In assessing the favorability of the cervix for vaginal delivery, one might confuse which cervix is leading to the uterus carrying the fetuses. This could lead to a diagnosis of an unfavorable cervix, leading to a cesarean section when a vaginal delivery would have been possible. Diagnostic modalities are not readily available at most district facilities and where available, patients may not be able to afford them. In low-resource settings such as ours, many cases likely go undetected due to poor health-seeking behavior and the unavailability of diagnostic equipment. This is particularly true in the largely rural populations of Ghana characterized by prevailing poverty and different forms of obstetric delays. This also implies that women have poor access to care, as seen in our patient, in whom poor health-seeking behavior likely prevented the diagnosis of uterus didelphys as a possible cause of prior miscarriages. For the current pregnancy, in which her level of antenatal care-seeking behavior was considered acceptable, a low index of suspicion and inadequate imaging led to the missed diagnosis of uterus didelphys. Her uterus didelphys went undiagnosed after two previous miscarriages and two 2D antenatal ultrasounds until she underwent a cesarean section for the current pregnancy.

In low-resource contexts, the possibility of few reported cases may be attributed to an interplay between having uncomplicated pregnancies or deliveries and the likelihood of missing a diagnosis due to diagnostic limitations. There may also be a low interest among obstetricians to report such rare cases. In the absence of any standard guidelines, the low rate of reporting makes it important to document clinicians´ experiences in managing such cases, particularly given the high incidence of poor outcomes of pregnancies in women with uterine malformations in general. Some researchers have recently called for the continuous publication of case reports pertaining to the management of twin gestation in uterine malformations to broaden the data available on such rare presentations [9]. Others have also recommended the maintenance of a global register of such cases in order to improve pregnancy outcomes and fetal survival [10]. It is in response to these concerted calls that we report this case. To the best of our knowledge, this is the first case report of twin gestation in uterus didelphys in Ghana, a lower-middle-income country. Our case highlights the fact that the diagnosis of uterus didelphys could potentially be missed in the antenatal and perinatal periods in resource-limited settings in which access to quality ultrasound and other essential diagnostic procedures is not always assured. To decrease the chances of missing such rare presentations, we advocate for further training of sonographers working in district facilities and rural areas in order to recognize them. It is also worth emphasizing the importance of improving the prenatal diagnostic capabilities of healthcare workers in antenatal clinics and labor wards. As no specific guidelines exist for such cases, the management of women with such uterine malformations is left to the discretion of the clinician and his/her clinical experience. Thus, consistent registration of such cases and studies aimed at formulating guidelines for the diagnosis and management of uterus didelphys and other uterine malformations by various tiers of health workers should be encouraged to achieve favorable maternal and fetal outcomes.

## Conclusion

We present a rare case of uterus didelphys with twin gestation, both babies in one uterus, which is rarely reported. Early identification can influence management in pregnancy and after delivery. Despite being a rare presentation, we wish to create awareness among health workers in rural and low-resource settings of such cases and highlight the need to improve prenatal diagnostic capabilities, as this is key to determining the mode of delivery and achieving favorable maternal and fetal outcomes.

## References

[1] Ture A, Gedafa L, Gure T, Teshome A (2020). Case report dicavitary twin pregnancy in undiagnosed uterus didelphys delivered by caesarean section. Ethiop Med J.

[2] Cyrille NN, Grégory A, Sandrine M, Étienne B, Junie M, Bertrand KO (2019). Septate uterus with cervical duplication and longitudinal vaginal septum: A two case series. Laparosc Endosc Robot Surg.

[3] Heinonen PK (2000). Clinical implications of the didelphic uterus: long-term follow-up of 49 cases. Eur J Obstet Gynecol Reprod Biol.

[4] Martínez-Beltrán M, Giménez J, Acién P (2012). Uterus Didelphys with Septate Cervix and Unilateral Endometrial Carcinoma: A Case Report. J Genit Syst Disord.

[5] Chan YY, Jayaprakasan K, Zamora J, Thornton JG, Raine-Fenning N, Coomarasamy A (2011). The prevalence of congenital uterine anomalies in unselected and high-risk populations: a systematic review. Hum Reprod Update.

[6] Saravelos SH, Cocksedge KA, Li T-C (2008). Prevalence and diagnosis of congenital uterine anomalies in women with reproductive failure: a critical appraisal. Hum Reprod Update.

[7] Fox NS, Roman AS, Stern EM, Gerber RS, Saltzman DH, Rebarber A (2014). Type of congenital uterine anomaly and adverse pregnancy outcomes. J Matern Fetal Neonatal Med.

[8] Arora M, Gupta N, Neelam, Jindal S (2007). Unique case of successful twin pregnancy after spontaneous conception in a patient with uterus bicornis unicollis. Arch Gynecol Obstet.

[9] Adams K, Minuzzo L (2019). Successful delivery of spontaneously conceived twins in a single horn of a bicornuate uterus: A case report. Case Rep Womens Health.

[10] Ahluwalia S, Kamble V, Patil S, Boricha B (2014). A unique case of successful twin pregnancy reaching term in a patient with uterus bicornis unicollis. Int J Reprod Contracept Obstet Gynecol.

